# The New Year

**Published:** 1866-01

**Authors:** 


### Editorial.

### THE NEW YEAR.

We extend to all our readers, a Happy New Year's greeting.

The year 1865, with all its mighty events, changes, and vicissitudes, is of the things that were. It is profitable, perhaps, for all to look back over this page of time and see how the account stands, whether the credit or debtor column is the heavier.

Many, on looking back over the record, will be surprised to find that it has been by them kept so illegibly. The life of many is so aimless, and accomplishes so little, that so far as they, or those with whom they are associated are concerned, no clear record is made upon time's page, or even upon the tablet of their memory.

Every one should live, in such a manner, as to be conscious of accomplishing good in the world,—that his life is a continual succession of good acts.

He who lives solely for himself, had better never been born, at least to human appearance. Whatever the record of the past may be, good or bad, clear or obscure, the question is now presented to us, What record shall we make this year?

Such thoughts as these, may have a very wide range, and extensive application, but they are just as clearly applicable, to particulars, as to generalities. To us, as professional men, and as dentists, they are applicable. Have we, each one in his particular sphere, during the year which has passed, accomplished all the good in his power, or are we not, many of us, all the time consciously working upon a plain far below that we might occupy? Have we all embraced, earnestly, all the opportunities presented to us, for our progress and elevation, or is there not rather too much disposition to plod along in the old routine way.

The circumstances and conditions for the progress and development of our profession, are now far more favorable than ever before.

The American mind has undergone a training, eminently calculated to strengthen and stimulate to more vigorous action.

There will in the future be a concentration of renewed energy, that will produce unparalleled results, in every department of human action; he who does not come up to this point, will be accounted a drone—at least comparatively.

Let those who imagine that they have made all the progress that it is possible to make, take heed lest they are left in the dim distance behind; old things are passing away, and all things are becoming new; a glorious day is dawning upon us, let all be prepared to enjoy its brightness.

